# Multimodal Study of *PRPH2* Gene-Related Retinal Phenotypes

**DOI:** 10.3390/diagnostics12081851

**Published:** 2022-07-31

**Authors:** Giulio Antonelli, Mariacristina Parravano, Lucilla Barbano, Eliana Costanzo, Matteo Bertelli, Maria Chiara Medori, Vincenzo Parisi, Lucia Ziccardi

**Affiliations:** 1 IRCCS—Fondazione Bietti, Via Livenza 3, 00198 Rome, Italy; giulio.antonelli@fondazionebietti.it (G.A.); lucilla.barbano@fondazionebietti.it (L.B.); eliana.costanzo@fondazionebietti.it (E.C.); vincenzo.parisi@fondazionebietti.it (V.P.); lucia.ziccardi@fondazionebietti.it (L.Z.); 2 MAGI’S LAB, Via Delle Maioliche 57/D, 38068 Rovereto, Italy; matteo.bertelli@assomagi.org (M.B.); chiara.medori@assomagi.org (M.C.M.); 3 MAGI EUREGIO, Via Maso Delle Pieve 60/A, 39100 Bolzano, Italy; 4 MAGISNAT, Atlanta Tech Park, 107 Technology, Parkway, Peachtree Corners, GA 30092, USA

**Keywords:** PRPH2, retinal dystrophy, novel variants, choroidal neovascularization, extensive chorioretinal atrophy, multimodal imaging, electroretinogram

## Abstract

*PRPH2* gene mutations are frequently found in inherited retinal dystrophies (IRD) and are associated with a wide spectrum of clinical phenotypes. We studied 28 subjects affected by IRD carrying pathogenic *PRPH2* mutations, belonging to 11 unrelated families. Functional tests (best-corrected visual acuity measurement, chromatic test, visual field, full-field, 30 Hz flicker, and multifocal electroretinogram), morphological retino-choroidal imaging (optical coherence tomography, optical coherence tomography angiography, and fundus autofluorescence), and clinical data were collected and analyzed. Common primary complaints, with onset in their 40s, were visual acuity reduction and abnormal dark adaptation. Visual acuity ranged from light perception to 20/20 Snellen. Visual field peripheral constriction and central scotoma were found. Chromatic sense was reduced in one third of patients. Electrophysiological tests were abnormal in most of the patients. Choroidal neovascular lesions were detected in five patients. Three novel *PRPH2* variants were found in four different families. Based on the present multimodal study, we identified seven distinct *PRPH2* phenotypes in 11 unrelated families carrying either different mutations or the same mutation, both within the same family or among them. Fundus autofluorescence modality turned out to be the most adequate imaging method for early recognition of this dystrophy, and the optical coherence tomography angiography was highly informative to promptly detect choroidal neovascularization, even in the presence of the extensive chorioretinal atrophy phenotype.

## 1. Introduction

Mutations in the peripherin-2 (*PRPH2*) gene are frequently found in inherited retinal diseases (IRD) [1,2]. This gene is located on chromosome 6p21.2 and is also known as retinal degeneration slow (RDS) gene. 

The gene product, the PRPH2 protein, is a member of the tetraspanin family, a transmembrane structural glycoprotein with an integral role in the formation and structure of both rod and cone photoreceptor outer segments [3,4]. The protein, containing four transmembrane domains and an intracellular domain, forms intramolecular disulfide bonds [5,6,7] and mediates assembly of peripherin-2/retinal outer segment membrane protein 1 (PRHP2/ROM1) tetramers into covalently linked higher-order complexes [7]. The formation of this protein complex is quite important for the functional activity of the protein, that is to create and maintain the rim region of rod discs and cone lamellae and to regulate disc size and alignment [7]. 

Although the mechanism of action of *PRPH2* gene alterations is still not completely understood [8], different related clinical pathological presentations have been described: pattern dystrophy (PD), multifocal pattern dystrophy simulating fundus flavimaculatus (PDSFF), macular dystrophy (MD), Stargardt disease (SD), retinitis pigmentosa (RP), adult-onset vitelliform macular dystrophy (AVMD), extensive chorioretinal atrophy (ECA) and central areolar choroidal dystrophy (CACD) [7,9,10,11,12].

This phenotypic heterogeneity makes the definition of the disease very challenging, for various reasons. The first one is that a transition from one clinical classification to another is possible as patients grow older; indeed, it has been reported that patients showing early and pure macular dystrophy phenotype will progress to a cone-rod or rod-cone dystrophy [7]. The second reason is the inter- and intra-familial phenotypical variability, even among family members carrying the same mutant allele [13,14], possibly due to other genetic modifiers, as ROM1, (in digenic RP), ABCA4 (typically in autosomal recessive SD), and RPE65 variants [8,15,16,17]. 

Because of the phenotype variability, the diagnosis is often delayed, and the real number of patients affected by *PRPH2*-related retinal dystrophy may be underestimated. 

Indeed, the prevalence of *PRPH2* disease is reported differently among countries, 10.3% in France, 9% in America, 9% in Italy, 5% in Japan, and 3.5% in North America [2,18,19,20,21]. Moreover, because of the reduced frequency out of Europe, a European ancestry has been suggested [21].

From the observation of several different IRD in our clinical practice, we focused on those patients with a confirmed known pathogenic mutation, and a possible novel one in the *PRPH2* gene, detected by a next generation sequencing (NGS) large genetic panel for MD, cone-rod dystrophy (CRD), and RP cases.

A retinal multimodal study was conducted on a cohort of *PRPH2* patients with the aim to describe the clinical characteristics of the spectrum phenotype and to unveil whether the presence of choroidal neovascularization (CNV) was relevant or could be considered an unusual associated feature, as in other IRD. 

## 2. Materials and Methods

All research procedures described in this work adhered to the tenets of the Declaration of Helsinki. The study protocol (NEU_01-2014) was approved by the local ethical committee (Comitato Etico Centrale IRCCS Lazio, Sezione IFO/Fondazione Bietti, Rome, Italy) and informed consent after full explanation of the procedures was obtained from each subject included in the study.

We retrieved from our IRD registry, all patients with a pathogenic mutation of the *PRPH2* gene and collected clinical and instrumental examinations performed during their visits. Data presented in the present study refer to the last visit.

All patients underwent best-corrected visual acuity (BCVA) measurement by the early treatment of diabetic retinopathy study (ETDRS) charts (Lighthouse Precision Vision, Woodstock, IL, USA) expressed in Snellen, chromatic test evaluated by Ishihara charts, slit-lamp fundus indirect ophthalmoscopy (with 90D Volk lens and dilated pupil by tropicamide 1% drops), kinetic visual field test by Goldmann perimeter (Haag-Streit, Bern, Switzerland), fundus autofluorescence (FAF) imaging, at 50° and 30°, by Spectralis (Heidelberg Engineering, Heidelberg, Germany), spectral domain optical coherence tomography (sdOCT), and where necessary, as a confirm for neovascular lesion, appropriate imaging as OCT-angiography (OCT-A) and/or fluoresceine angiography (FA). Full-field electroretinogram (ffERG) (by Retimax CSO, Firenze, Italy) and multifocal electroretinogram (mfERG) (using VERIS Clinic TM version 4.9; Electro-Diagnostic Imaging, San Mateo, CA, USA), with a multifocal stimulus consisting of 61-scaled hexagons, were recorded in accordance with the standards of the International Society for Clinical Electrophysiology of Vision [22,23,24] by using Dawson, Trick, and Litzkow (DTL) electrodes. We performed ffERG after 10 min of dark adaptation (flash 1J at 1 Hz), 30 Hz flicker ERG after 10 min of light adaptation and the mfERG, whose peak-to-peak response amplitude density (RAD), was measured in nanoVolt/degree^2^ (nV/d^2^) between the first negative peak (N1) and the first positive peak (P1), as described in our previous works [25,26,27].

### Genetic Testing

Genetic testing was performed at MAGI’s laboratory (MAGI’S Lab, Rovereto, Italy, and MAGI Euregio, Bolzano-Bozen, Italy) from salivary samples, after genetic counseling to reconstruct the family pedigree and after obtaining informed consent and explaining the characteristics of a genetic test. We assumed the mode of inheritance as autosomal dominant if two generations or more were affected; autosomal recessive if there was parental consanguinity or siblings from normal parents were affected; patients not reporting parental consanguinity and not having any evidence of other affected family members were defined as “sporadic”. 

The patients were tested between 2014 and 2021 via targeted NGS performed on a MiSeq personal sequencer (Illumina, San Diego, CA, USA), using panels that include genes associated with RP, MD, and pattern dystrophy. Family members of the proband were analyzed only for the variants already known and for genes modifier, as associated with the phenotype in the first family member tested with PCR. The pathogenicity of variants was evaluated according to the American College of Medical Genetics and Genomics (ACMG) guidelines [28].

## 3. Results

### 3.1. Clinical Findings

Demographic and functional data of the study cohort are reported on Table 1. 

From a total of 63 patients belonging to 11 unrelated families, we found 34 patients carrying a *PRPH2* mutation. Among them, two subjects, deceased after the genetic test, (done for completing the family segregation study) and four subjects were unavailable to come to our center to be studied. Thus, we had the opportunity to collect clinical and genetic data from 28 affected subjects. All these latter patients had some visual complaints or some clinical findings typical of bilateral and symmetric IRD. 

All the family’s pedigrees are available in Figure S1 in Supplementary Material. 

The autosomal dominant inheritance pattern was verified in 6 out of 11 families, the remnants were defined as sporadic.

Our cohort included 14 females and 14 males. The age of the patients ranged from 37 to 79 years with a mean (±SD) of 58 ± 1235 and the mean age of onset of symptoms was 41 ± 1283 years old, similar to data already reported in previous studies [7,9,20]. BCVA of the patients ranged from light perception to 20/20 Snellen. Common primary complaints were reduction in VA (10 patients, 35%), difficulty in dark adaptation (7 patients, 25%), metamorphopsia and photophobia (both 5 patients, 17%) in accordance with other previous reports [7,9]. 

Of note, six subjects (21%) had no symptoms, and they were detected only because of sibilants of other patients. 

The most frequent visual field defects were peripheral constriction (8 patients, 28%), central scotoma (7 patients, 25%) and ring scotoma (4 patients, 14%); in a small percentage of patients no abnormalities were detected at the visual field (4 patients, 14%). We found abnormal chromatic test in nine patients (32%) of our cohort, data not reported in other studies, except for one reported patient [29]. The a-b wave amplitude of the scotopic ffERG and the amplitude of the 30 Hz flicker ERG were reduced similarly in the majority of patients (22 patients, 78%). The mfERG RAD was found reduced between 0–20 degrees in 15 patients (53%), whereas localized dysfunction was found within 0–5 degrees in another six patients (21%) and reduced RAD within 10–20 degrees was found in only two patients (7%); mfERG RAD was found normal in four patients (14%) and in one patient this data was not available. 

**Figure 1:**
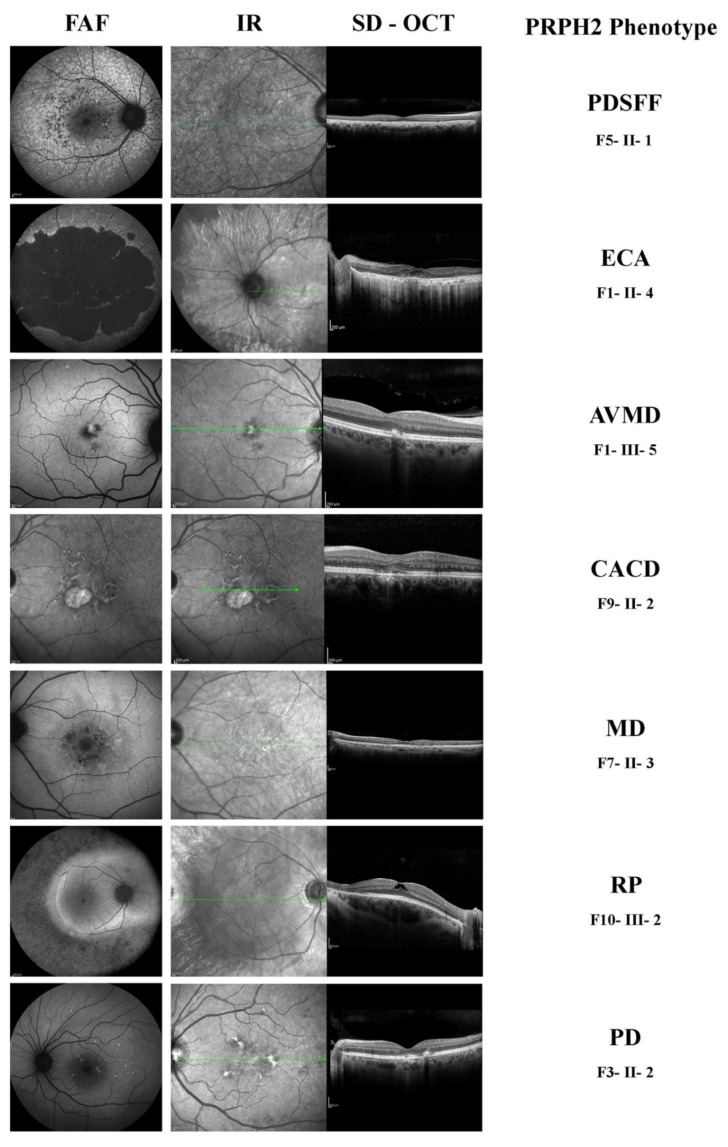
Inter-familiar genetic variability of *PRPH2*-related retinal dystrophy. Fundus autofluorescence (FAF), Infra-red (IR) and spectral-domain optical coherence tomography (SD-OCT) acquisitions of different *PRPH2* phenotypes due to different variants of the same gene in different unrelated families. PD, pattern dystrophy; PDSFF, multifocal pattern dystrophy simulating fundus flavimaculatus; MD, macular dystrophy; RP, retinitis pigmentosa; AVMD, adult-onset vitelliform macular dystrophy; ECA, extensive chorioretinal atrophy; CACD, central areolar choroidal dystrophy.

As above mentioned, and based on previous clinical reports of *PRPH2* families, we identified seven different phenotypes associated with *PRPH2* mutation in our cohort. Patients displayed clinical features varying from RP to MD. For instance, we identified a total of five patients (17%) with autosomal dominant RP (ADRP), two patients with MD (7%), three patients (10%) with AVMD, six patients (21%) with PD, two patients (7%) with CACD, four with ECA (14%), and six patients (21%) with PDSFF. Fundus aspect and retinal morphological features detected by SD-OCT and FAF are reported in Table 2. Representative examples of the seven different phenotypes are illustrated in Figure 1. 

An unusual feature already discussed and reported in the literature [20,30,31] was the presence of monocular CNV in five affected patients (17%) in our group presenting with different phenotypes (PD, CACD, ECA). 

### 3.2. Genetic Findings

We studied 11 families with seven distinct *PRPH2* genetic variants. Among these, we found three novel *PRPH2* variants not previously reported: the same variant c.734dup; p.(Trp246Valfs*55) was found in two unrelated families (family 4 and family 8), the variant c.903del; p.(Ser301Argfs*23) was found in family 10 and another one c.742C > A; p.(Arg248Ser) in family 11. Another already known variant, c.499G > A; p.(Gly167Ser), was found in four unrelated families (family 1, 3, 5 and 9); moreover in family 2 the variant c.290G > A; p.(Trp97*) was found, in family 6 the variant c.136C > T; p.(Arg46*), and in family 7 the variant c.623G > A; p.(Gly208Asp), these last four variants were previously reported. 

Concerning the modifier genes, we found a total of five variants (four on ABCA4 gene and one on ROM1 gene) in five patients of five unrelated families.

Genotype data including a detailed list of *PRPH2* variants, genetic modifiers and correlated clinical diagnosis are presented on Table 3. 

## 4. Discussion

We performed a retinal multimodal study in a cohort of patients carrying causative mutations of the *PRPH2* gene that, to our knowledge, represents at the present time the biggest study in Italy.

The study was conducted in a cohort of 28 *PRPH2* patients with the aim of describing the clinical variability of the wide spectrum phenotype, which was classified in seven main types. The present work also described the presence of monolateral choroidal neovascularization in five patients, as an unexpected but relevant feature, unusually associated with other IRD. 

Among the *PRPH2* variants found to be pathogenic in our cohort, we also described three novel mutations, one of which was found in members of two unrelated families.

### 4.1. Phenotype-Genotype Variability of PRPH2 Disease Related Spectrum 

We found the clinical diagnosis and the classification of the disease considering the variable clinical spectrum at presentation to be challenging. Despite it appearing that only one gene was involved in the pathogenesis of the disease, the retinal dystrophy presented in almost seven different phenotypes involving the peripheral retina (i.e., retinitis pigmentosa, extensive chorio-retinal atrophy, pattern dystrophy-simulating fundus flavimaculatus), or the central macula (i.e., macular dystrophy, AVMD, CACD, pattern dystrophy), as illustrated in Figure 1. 

**Figure 2:**
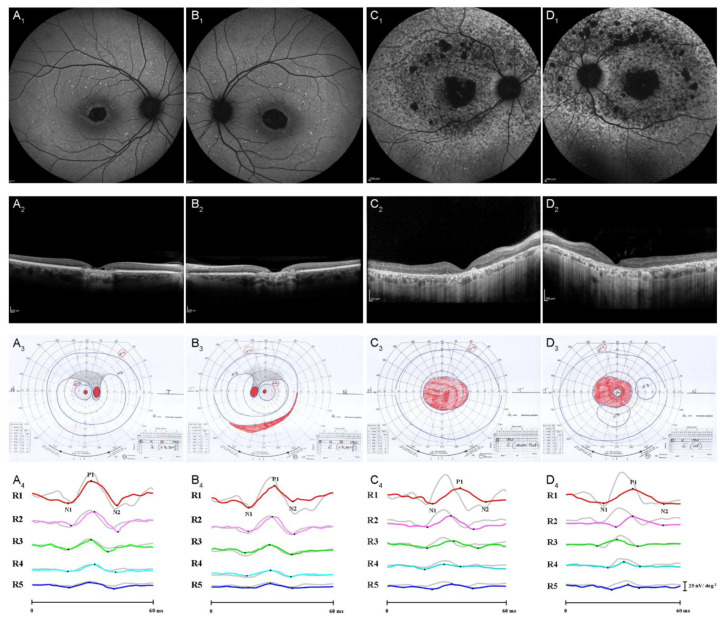
Inter-familiar phenotypic variability of *PRPH2*-related retinal dystrophy. Column **A** and **B** showing Right eye and left eye of F4-III-3 (52 years old at time of examination), Column **C** and **D** showing right eye and left eye of F8-II-1 (56 years old at time of examination). On line **A_1_**–**D_1_** are displayed fundus autofluorescence (FAF), on line **A_2_**–**D_2_** are displayed spectral-domain optical coherence tomography (SD-OCT), On line **A_3_**–**D_3_** are displayed Goldmann visual field test and on line **A_4_**–**D_4_** are displayed multifocal electroretinogram (mfERG) ring (R) traces overlayed by control trace. Different phenotypes in different families carrying the same mutation in *PRPH2* gene are displayed.

Indeed, we observed in our cohort that phenotype variability was present:

(1) in unrelated families carrying different mutations (inter-familiar genetic variability), as expected from already reported studies [7,13,14] and depicted in Figure 1.
(2) in different unrelated families carrying the same mutation (inter-familiar phenotype variability), as reported in Figure 2.

The relationships between the clinical features and genetic variants are still unclear because the same genetic variant can affect rods and cones differently. [3,7] Therefore, without consistent genotype–phenotype correlations, the accepted view is that a single mutation in *PRPH2* may cause a spectrum of phenotypes, impacting on both the central photopic system and peripheral scotopic cellular elements. In other reports it is evidenced that many genetic variants are mostly found in the D2 loop [11,20], which is critical for protein–protein interactions. In agreement, we found that most of our patients have a mutation in this domain, except for family 2 (Trp97*), family 6 (Arg46*), and family 10 (Ser301A).

We found it valuable to acquire FAF imaging for all patients. By analyzing the 50° and 30° images (Figure 1) we classified the *PRPH2* retinal dystrophy spectrum and found common characteristics of seven different patterns. To explain the uncommon and variable presentation of this monogenic disease, as already hypothesized, we accounted for other factors such as genetic background, genetic modifiers, and/or environmental factors that may affect phenotypes and outcomes [7,8,15,16,17]. As recently reported, it is likely that also mRNA and protein expression levels and/or post transcriptional regulatory mechanisms are intermediate factors between gene expression and clinical phenotypes [32,33]. 

Of interest, we documented different presentations in unrelated families carrying the same identical gene mutation either already described, as found in families 1, 3, 5, and 9 (c.499G > A) or novel (c.734dup), as reported in families 4 and 8 (as illustrated in Figure 2). About the cases with the c.734dup mutation, the phenotype variability could be given by the genetic modifiers *ABCA4*, which resulted, however, differently mutated in both families, and could influence the prognosis.

**Figure 3:**
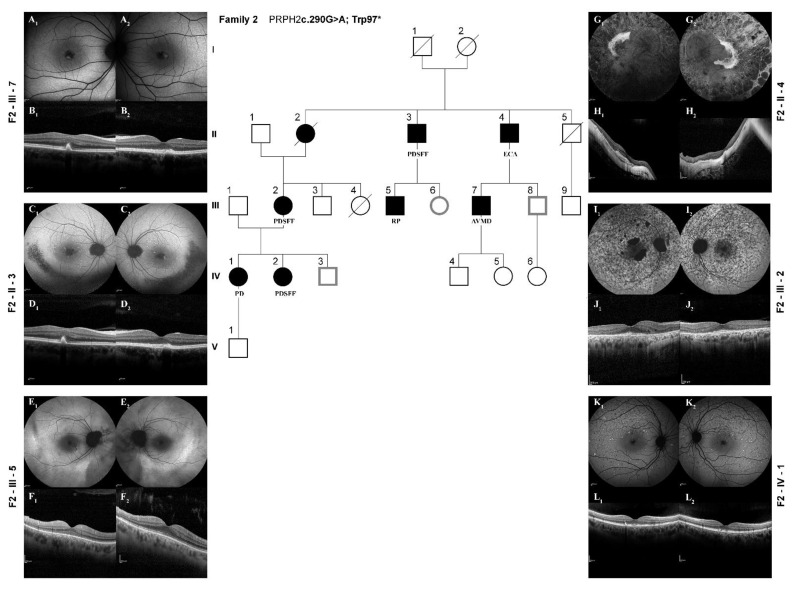
Intra-familiar variability of *PRPH2*-related retinal dystrophy. Fundus autofluorescence (FAF) and spectral-domain optical coherence tomography (SD-OCT) acquisitions in patients belonging to the same large pedigree (**Family 2**), thus harboring the same *PRPH2* mutation and presenting with different phenotypes. (**A1**,**A2**,**B1**,**B2**): F2-III-7 FAF and OCT (44 years old at time of examination), AVMD; adult-onset vitelliform macular dystrophy; (**C1**,**C2**,**D1**,**D2**): F2-II-3 FAF and OCT (70 years old at time of examination), PDSFF, multifocal pattern dystrophy simulating fundus flavimaculatus; (**E1**,**E2**,**F1**,**F2**): F2-III-5 FAF and OCT (39 years old at time of examination), RP, retinitis pigmentosa, (**G1**,**G2**,**H1**,**H2**): F2-II-4 FAF and OCT (79 years old at time of examination) ECA, extensive chorioretinal atrophy; (**I1**,**I2**,**J1**,**J2**): F2-III-2 FAF and OCT (66 years old at time of examination), PDSFF; (**K1**,**K2**,**L1**,**L2**): F2-IV-1 FAF and OCT (41 years old at time of examination), PD, pattern dystrophy.

In addition to this interfamilial variability, an interesting feature that we found is the important intrafamilial variability identified in family 2, where the same identical mutation (c.290G > A) produced five different presentations (PD, PDSFF, ECA, ADVM, and ADRP), as illustrated in Figure 3. 

This example enabled us to think that there could be unidentified factors, more than the classic genetic one, that can influence the translation of the phenotype. Moreover, we could further observe a follow-up of 6 years of a member of Family 2 (F2-III-7) showing AVMD (Figure 3A,B) feature at the onset of the disease (age of 38 y/o) with a subsequent reabsorption of the vitelliform lesion without progression of the outer retinal layer to atrophy, as usually expected [34], which can justify the good BCVA at the present.

### 4.2. Unreported Clinical Functional and Morphologic Characteristics of PRPH2-Related Disease 

Although previous authors have tried to find out some distinctive traits of this spectrum disorder, as above-mentioned, this is one of the most variable IRD. In addition to that already reported in other IRD, we found an alteration of the chromatic sense, which was interestingly reduced only in those *PRPH2* patients with a reduction of BCVA. This observation was not confirmed by Sonia H et al. [35] who reported an alteration in chromatic perception even with good BCVA, however the study cohort was made of patients with only best maculopathy. 

On the functional assessment by electroretinographic signals, Rola Ba-Abbad et al. [36] described a case series of six patients (51.6 ± 11.86 years old), all with *PRPH2* mutations but with different retinal involvement, all with an electronegative electroretinogram waveform (full field scotopic and photopic ERG), later confirmed only by one more report [37]. In our cohort, which appears slightly older (58 ± 12.35 years old) none of our patients displayed an electronegative ERG. It is likely that the electronegative ERG is not pathognomonic of *PRPH2* related dystrophy, contrary to that previously hypothesized [36]. In addition, since we found similarly reduced signals derived from scotopic and photopic cellular systems of the outer retina, we were not able to establish whether *PRPH2* related disorder mimics a cone-rod or a rod-cone dystrophy

Concerning the electrofunctional assessment of the macular region, we were able to record mfERG in almost all subjects and found reduced RAD in the majority [17]. Interestingly of these, eight had preserved BCVA (20/20 Snellen) and only slightly macular involvement evident at the SDOCT. As far as we know, there are only a few mfERG studies [38,39] in patients with this phenotype and one of these included only four patients with evident macular involvement. Our finding of reduced mfERG responses describes a dysfunction of photoreceptors and bipolar cells in this retinal degeneration. 

Of interest, the presence of CNV was relevant in our cohort. A recent report by Yousra Falfoul et al. [40] assessed a frequent macular involvement with CNV in RP patients, enough to consider the research of *PRPH2* gene mutation, when a CNV is observed. In agreement with this observation, we found monocular CNV in five subjects, presenting with RP and PD phenotypes, as already reported [20,30,31], and associated with ECA phenotype, not previously reported (F3-I-2, Figure 1 and F2-II-4, Figure 3). The follow-up of CNV found in our cohort was not complete, as only one patient underwent anti-vascular endothelial growth factor (VEGF) intravitreal injection in our center.

Our findings are relevant because, as previously suggested [40], all *PRPH2* patients displaying PD or RP phenotypes should be followed by SD-OCT and OCTA for the possibility of developing CNV as a complication of the disease. This alert should be extended to *PRPH2* patients displaying ECA features. This agrees with a previous OCTA study that highlighted the importance of assessing vascular retino-choroidal alterations, such as the already described increase in the size of the foveal avascular zone (FAZ) at the superficial vascular plexus (SVP) or deep capillary complex (DCP), appreciable in *PRPH2* patients presenting a phenotype of CACD and ECA [12]. 

Concerning the imaging, FAF was revealed as a useful tool to detect and observe the seven phenotypes of this IRD and especially PDSFF. This phenotype was characterized by the appearance of a speckled point of hypo- and hyper-autofluorescence in the posterior pole and beyond the vascular arcades (see Figure 1 and Figure 3) Only the thorough acquisition of the FAF modality in all patients allowed the seven patterns (see Figure 1) to be distinguished and the *PRPH2* IRD to be easily differentiated from any others that could have been misdiagnosed using only the SD-OCT scans. All this let us propose the FAF modality as the most appropriate morphological method to categorize the retinal prototypical characteristics of the *PRPH2* disease spectrum, especially the PDSFF type which presents peculiar abnormalities along the vascular arcades and otherwise not detectable easily by SD-OCT. In agreement with this observation, a previous study [41] described that quantitative fundus autofluorescence (qAF) may help to distinguish patients with *PRPH2* gene mutations. In this group, qAF values were lower than in patients with *ABCA4* gene mutations but higher with respect to control subjects [22]. 

## 5. Conclusions

The identification of *PRPH2* IRD is challenging, and the rate of affected population may be underestimated because of the clinical variability of the different phenotypes, and thus the numerous misdiagnosed cases with limited access to genetic testing. We reported three novel *PRPH2* variants: the c.734dup associated with PD, PDSFF and CACD in two unrelated families, the c.903del associated with RP, and the c.742C > A associated with PD in another two distinguished families. We observed new electrophysiological features of the *PRPH2* spectrum phenotypes, consisting of an impairment of the mfERG, even in those patients with preserved BCVA and only slightly macular SD-OCT alterations. We propose in *PRPH2* patients FAF modality as the most suitable and accessible imaging method to identify the disease phenotypes and OCTA acquisition to promptly detect CNV, even in patients with ECA phenotype, and for a correct diagnosis, advocating the programing of a correct follow up for appropriate management of this complication. 

## Figures and Tables

**Table 1:** Demographic and functional characteristics of *PRPH2* patients.

Patient	Gender	Age at Examination	Age at Disease Onset	Symptoms at Onset	BCVA at Last Visit RE; LE	Chromatic Test (Ishihara Charts) in OU	Visual Field in OU	Scotopic ffERG a-b Wave Amplitude in OU	Flicker 30 Hz Amplitude in OU	mfERG RAD in OU
F1-III-8	F	45	29	metamorphopsia	20/20; 20/20	normal	blind spot enlargement (I/4)	normal	reduced	normal in all rings
F1-II-4	M	74	66	visual acuity reduction	Counting fingers; 20/32	pathologic	central scotoma (III/4)	reduced	reduced	reduced in all rings
F1-II-3	M	68	60	visual acuity reduction	20/400; 20/40	pathologic	central scotoma (V/4)	reduced	reduced	reduced in all rings
F1-III-5	M	44	45	visual acuity reduction, metamorphopsia	20/20; 20/20	NA	normal	normal	normal	normal in all rings
F2-III-7	M	44	38	photophobia, difficulty dark adaptation	20/20; 20/20	normal	ring scotoma (I/3)	reduced	reduced	normal in all rings
F2-III-5	M	39	36	photophobia	20/20; 20/20	normal	peripheral restriction (I/4)	reduced	reduced	reduced in all rings
F2-II-4	M	79	35	visual acuity reduction and photophobia	light perception; 20/25	pathologic	peripheral restriction (V/4)	reduced	reduced	NA
F2-II-3	M	70	30	photophobia	20/20; 20/20	normal	blind spot enlargement (I/4)	reduced	reduced	reduced in all rings
F2-IV-2	F	37	34	difficulty dark adaptation	20/20; 20/20	normal	normal	reduced	normal	reduced R1 and R3 in OU
F2-IV-1	F	41	33	metamorphopsia	20/20; 20/20	normal	peripheral restriction (I/2)	normal	normal	reduced in R1-R2
F2-III-2	F	66	30	metamorphopsia	20/20; 20/20	pathologic	ring scotoma (III/4)	reduced	reduced	reduced in all rings
F3-I-2	F	73	40	photophobia	counting fingers; 20/50	pathologic	NA	reduced	reduced	reduced in all rings
F3-II-1	F	50	/	casual finding	20/20; 20/20	normal	normal	normal	normal	normal in all rings
F3-II-2	M	53	42	visual acuity reduction	20/20; 20/20	normal	blind spot enlargement (I/2)	normal	normal	reduced in all rings
F4-III-1	F	57	/	casual finding	20/28; 20/20	pathologic	NA	normal	normal	reduced R1-R3
F4-III-3	M	52	43	difficulty dark adaptation, visual acuity reduction	20/200; 20/40	pathologic	peripheral restriction (I/4)	reduced	reduced	reduced in all rings
F5-II-1	M	69	65	casual finding	20/20; 20/20	normal	central scotoma (I/4)	reduced	reduced	reduced R2-R5
F6-III-1	M	60	30	casual finding	20/28; 20/28	normal	NA	reduced	reduced	reduced in all rings
F7-II-3	F	60	/	casual finding	20/20; 20/20	normal	ring scotoma (I/4)	normal	normal	reduced R1-R2
F8-II-1	F	56	40	difficulty dark adaptation,	20/50, 20/66	normal	central scotoma (I/2)	normal	normal	reduced R1
F9-II-1	F	70	20	visual acuity reduction and photophobia	20/32; 20/200	normal	central scotoma and peripheral restriction (I/4)	reduced	reduced	reduced R1-R2
F9-II-6	F	74	63	visual acuity reduction	light perception; 20/40	pathologic	central scotoma and peripheral restriction (III/4)	reduced	reduced	reduced in all rings
F9-II-7	M	62	55	casual finding	20/20; 20/20	normal	normal	normal	normal	reduced in all rings
F9-II-5	F	63	40	difficulty dark adaptation,	20/200; 20/32	pathologic	central scotoma (III/4)	normal	normal	reduced in all rings
F9-II-4	M	65	40	difficulty dark adaptation, metamorphopsia	20/63; 20/25	normal	ring scotoma (I/4)	normal	normal	reduced in all rings
F9-II-2	m	68	50	visual acuity reduction	20/400; 20/400	pathologic	peripheral restriction (V/4)	reduced	reduced	reduced in all rings
F10-III-2	F	37	16	difficulty dark adaptation,	20/20; 20/32	normal	peripheral restriction (I/3)	reduced	reduced	reduced R3-R5
F11-II-1	F	48	45	visual acuity reduction	20/20. 20/20	normal	NA	normal	normal	reduced in all rings

M, male; F, female; RE, right eye; LE, left eye; NA, not available; OU, both eyes; RAD, response amplitude density, I/1, I/2, I/3, I/4, III/4, V/4 refers to the kinetic visual field isopters tested.

**Table 2:** Morphological retinal aspect of *PRPH2* patients.

Patient	Fundus Aspect	Phenotype	FAF	SD-OCT	Evidence of CNV
F1-III-8	Simil-flecks lesions in mid-periphery along vascular arcades	PDSFF	Macular hypo-AF with speckled hyper-AF in the macular and mid-periphery associated with hypo-AF due to plaques atrophy in the mid-periphery	Hyper-reflective deposit above the RPE in the parafoveal region. EZ and ELM preservation in foveal and parafoveal region	No
F1-II-4	Diffuse chorioretinal atrophy, small trophic area in fovea in LE	ECA	Hypo-AF at the atrophic area extended in macular region and mid-periphery, involving the optic disc, speckled hyper-AF in the mid-periphery	Vitreo-macular adhesion. Disruption of the EZ and ELM in the parafoveal area with sparing of foveal region	No
F1-II-3	Chorioretinal atrophy with pigment dispersion along vascular arcades	ECA	Macular hypo-AF with speckled hyper-AF in the mid-periphery associated with hypo-AF due to plaques atrophy in the macula and mid-periphery	Vitreo-macular adhesion. Outer retinal atrophy of the macular region and choroidal hyper-reflectivity by window defect at the posterior pole and rarefaction of EZ and ELM and ORT in parafoveal region in RE. Disruption of the EZ and ELM in the parafoveal area with partial sparing of foveal region in LE	No
F1-III-5	Slight rehash in macula	AVMD	Parafoveal hyper-AF in RE. Macular hypo-AF with speckled hyper-AF in the macula in LE	Hyper-reflective deposit above the RPE in the foveal and parafoveal region	No
F2-III-7	Yellowish stippling in the periphery	AVMD	Macular hypo-AF with speckled hyper-AF in the mid-periphery	Hyper-reflective deposit above the RPE in the foveal and parafoveal region	No
F2-III-5	Slightly rehash in macula in LE	RP	Normal-AF of macula and mid-periphery in RE. Speckled hyper-AF in peripapillary region in LE	Normal profile and reflectivity of the inner and outer retinal layers and of RPE-CC complex	No
F2-II-4	Peripapillary chorioretinal atrophy with mid- and peripheral dystrophy	ECA	Macular hypo-AF with hyper-AF island in the parafoveal region Hypo-AF due to plaques atrophy in the mid-periphery	Foveal hyper-reflective lesion with ORT due to MNV scar n RE. Outer retinal atrophy of the macular region and choroidal hyper-reflectivity by window defect at the posterior pole in LE	CNV in RE
F2-II-3	Lipofuscin deposits in macula	PDSFF	Macular hypo-AF with speckled hyper-AF in the mid-periphery and granular hypo-AF in one sector (inferior) of the peripheral region	SDD/reticular pseudodrusen with rarefaction of EZ and ELM in foveal and parafoveal region	No
F2-IV-2	Many points of altered pigmentation at the posterior pole and outside vascular arcades	PDSFF	Macular hypo-AF with speckled hyper-AF and hyper-AF flecks at the posterior pole and mid-periphery	SDD/reticular pseudodrusen with rarefaction of EZ in foveal and parafoveal region	No
F2-IV-1	Small lipofuscin deposit near the fovea	PD	Focal hyper-AF in the parafoveal region	SDD/reticular pseudodrusen with rarefaction of EZ in foveal and parafoveal region	No
F2-III-2	Macular atrophy and altered pigmentation in the periphery	PDSFF	Macular hypo-AF with speckled hyper-AF and hyper-AF flecks at the posterior pole and mid-periphery in OU, hypo-AF due to plaques atrophy in the parafoveal regions in RE	Disruption of the EZ and ELM in the parafoveal area with sparing of foveal region in OU. In RE area of retinal atrophy in parafoveal region	No
F3-I-2	Diffuse areas of chorioretinal atrophy at the posterior pole and in mid periphery	ECA	Hypo-AF due to plaques atrophy in the macula and mid-periphery (RE > LE), associated with macular hypo-AF with speckled hyper-AF in the mid-periphery	Outer retinal atrophy of the macular region and choroidal hyper-reflectivity by window defect at the posterior pole in RE. Hyper-reflective deposit above the RPE in the foveal region followed by outer retinal atrophy of the macular region in LE	CNV in LE
F3-II-1	Lipofuscin deposits in LE	PD	Macular hypo-AF in macular region in BE with focal hyper-AF in the parafoveal region in LE	SDD/reticular pseudodrusen with rarefaction of EZ in parafoveal region	No
F3-II-2	Lipofuscin deposits with RPE rehash in macula	AVMD	Macular hypo-AF with hyper-AF flecks at the posterior pole	SDD/reticular pseudodrusen with rarefaction of EZ in parafoveal region	No
F4-III-1	SlightRPE rehash in macula	PD	Focal hyper-AF in the parafoveal region in RE	SDD/reticular pseudodrusen with rarefaction of EZ in foveal and parafoveal region	No
F4-III-3	Stippling outside vascular arcades	PDSFF	Macular hypo-AF at the atrophic macular area with speckled hyper-AF and hyper-AF flecks at the posterior pole and mid-periphery	Outer retinal atrophy of the macular region with choroidal hyper-reflectivity by window defect. Disruption of the EZ and ELM in the parafoveal region	No
F5-II-1	Stippling inside and outside vascular arcades	PDSFF	Macular hypo-AF with speckled hyper-AF and hyper-AF flecks at the posterior pole and mid-periphery	Disruption of the EZ and ELM in the parafoveal area with sparing of foveal region	No
F6-III-1	Pigment dispersion in the periphery	RP	Macular hypo-AF in macular region and granular hypo-FA in the mid-periphery	ERM, Disruption of the EZ and ELM in the macular and extramacular region (out of the posterior pole)	No
F7-II-3	Macular dystrophy	MD	Macular hypo-AF with speckled hyper- and hypo-AF at the posterior pole	Disruption of the EZ and ELM in the parafoveal area with sparing of foveal region SDD/reticular pseudodrusen with rarefaction of EZ in parafoveal region.	No
F8-II-1	Chorioretinal atrophy in macula with peripheral rehash	CACD	Macular hypo-AF at the atrophic macular area with speckled hyper-AF and hyper-AF flecks at the posterior pole and mid-periphery	Disruption of the EZ and ELM limited to the foveal region with outer retinal atrophy of the macular region and choroidal hyper-reflectivity by window defect	No
F9-II-1	RPE rehash in macula	RP	Normal-AF of macula and mid-periphery in RE. Speckled hyper-AF in peripapillary region in LE	Normal profile and reflectivity of the inner and outer retinal layers and of RPE-CC complex	No
F9-II-6	Pigment dispersion in the periphery, fibrotic scar in RE	MD	Macular hypo-AF with speckled hyper-AF and hyper-AF flecks at the posterior pole and mid-periphery	Disruption of the EZ and ELM in the parafoveal area with sparing of foveal region. SDD/reticular pseudodrusen with rarefaction of EZ in parafoveal region	No
F9-II-7	Pigment dispersion in the periphery	RP	Normal-AF of macula and mid-periphery in RE. Speckled hyper-AF in peripapillary region in LE	Normal profile and reflectivity of the inner and outer retinal layers and of RPE-CC complex	No
F9-II-5	Rehash of RPE in macula	PD	Hypo-AF due to fibrotic plaque in RE, focal hyper-AF in the parafoveal region	SDD/reticular pseudodrusen with rarefaction of EZ in foveal and parafoveal region, foveal hyper-reflective lesion due to CNV scar in RE	CNV in RE
F9-II-4	Rehash of RPE in macula	PD	Focal hyper-AF in the parafoveal region	SDD/reticular pseudodrusen with rarefaction of EZ in foveal and parafoveal region, lifting of RPE in LE	CNV in LE
F9-II-2	Rehash of RPE in macula and MNV in RE	CACD	Macular hypo-AF with hyper-AF in the foveal region in RE. Hypo-AF due to plaques atrophy in LE	Foveal hyper-reflective lesion with ORT due to MNV scar n RE. Disruption of the EZ and ELM limited to the foveal region with outer retinal atrophy and choroidal hyper-reflectivity by window defect in LE	CNV in RE
F10-III-2	Stippling of the posterior pole	RP	Hyper-AF ring that delineates the posterior pole with granular hypo-FA in the mid-periphery	Disruption of the EZ and ELM in the extramacular region (out of the posterior pole)	No
F11-II-1	Rehash of RPE in macula	PD	Focal hyper-AF in the parafoveal region	SDD/reticular pseudodrusen with rarefaction of EZ in foveal and parafoveal region.	No

SD-OCT, spectral domain optical coherence tomography, CNV, choroidal neovascularization; FAF, fundus autofluorescence; RPE, retinal pigmented epithelium; EZ, ellipsoid zone; ELM, external limiting membrane; ORT, outer retinal tubulations; SDD, subretinal drusenoid deposits, RPE-CC, retinal pigmented epithelium choriocapillaris complex; OU, both eyes; ERM, epiretinal membrane, RE, right eye; LE, left eye.

**Table 3:** Genotype and phenotype data of *PRPH2* cohort.

Family	*PRPH2* Gene Mutation	Inheritance	Clinical Significance	Mutation Type	Accession Number	Global Allele Frequency	Genetic Modifiers	Phenotypes of Our Patients
Family 1 (4 patients)	NM_000322.4: c.499G > A; p.(Gly167Ser)	AD	Pathogenic *	Missense	rs527236098	ƒ = 0.00000756	None	F1-III-8 PDSFF F1-II-3,4 ECA F1-III-5 AVMD
Family 2 (8 patients)	c.290G > A; Trp97*	AD	Pathogenic ^	Nonsense	/	/	None	F2-III-7 AVMD F2-II-4 ECA F2-II-3 PDSFF F2-IV-2 PDSFF F2-IV-1 PD F2-III-2 PDSFF F2-III-5 RP
Family 3 (3 patients)	NM_000322.4: c.499G > A; p.(Gly167Ser)	AD	Pathogenic *	Missense	rs527236098	ƒ = 0.00000756	None	F3-II-1 PD F3-II-2 AVMD F3-I-2 ECA
Family 4 (2 patients)	NM_000322.4: c.734dup; p. (Trp246Valfs*55)	AD	Pathogenic	Frameshift	/	/	ABCA4 c.5882G > A; Gly1961Glu	F4-III-1 PD F4-III-3 PDSFF
Family 5 (1 patient)	NM_000322.4: c.499G > A; p.(Gly167Ser)	/	Pathogenic *	Missense	rs527236098	ƒ = 0.00000756	None	F5-II-1 PDSFF
Family 6 (1 patient)	NM_000322: c.136C > T; p.(Arg46*)	/	Pathogenic §	Missense	rs139185976	ƒ = 0.0000159	None	F6-III-1 RP
Family 7 (1 patient)	NM_000322.5: c.623G > A; p.(Gly208Asp)	/	Pathogenic #	Missense	rs139185976	ƒ = 0.0000477	PROM1 Nonsense rs780697796c c.436C > T Arg146*	F7-II-3 MD
Family 8 (1 patient)	NM_000322.5; c.734dup; p. (Trp246Valfs*55)	/	Pathogenic	Frameshift	Unknown	/	ABCA4; c.514G > A; Gly172Ser: Missense rs61748532; AR	F8-II-1 CACD
Family 9 (7 patients)	NM_000322.4: c.499G > A; p.(Gly167Ser)	AD	Pathogenic *	Missense	rs527236098	ƒ = 0.00000756	ABCA4; c.5603A > T; Asn1868Ile; Missense; rs1801466; AR	F9-II-1,7 RP F9-II-6 MD F9-II-4,5 PD F9-II-2, CACD
Family 10 (1 patient)	NM_000322.5 c.903del; p. (Ser301ARGfs*23)	AD	Likely pathogenic	Frameshift	Unknown	/	ABCA4; c.6148G > C; Val2050Leu; Missense; rs41292677; AR	F10-III-2 RP
Family 11 (1 patient)	NM_000322: c.742C > A; p.(Arg248Ser)	/	Likely pathogenic	Missense	Unknown	/	None	F11-II-1 PD

PD, pattern dystrophy; PDSFF, pattern disease simulating fundus flavimaculatus; RP, retinitis pigmentosa; CRD, cone-rod dystrophy; AVMD, adult-onset vitelliform macular dystrophy; ECA, extensive chorioretinal atrophy; CACD; central areolar choroidal dystrophy. *: Testa, F.; Marini, V.; Rossi, S.; E, Interlandi.; Nesti, A.; Rinaldi, M.; Varano, M.; Garré, C.; Simonelli, F. A novel mutation in the RDS gene in an Italian family with pattern dystrophy, British Journal of Ophthalmology **2005**, 89, 1066–1068. #: Kohl, S.; Christ-Adler, M.; Apfelstedt-Sylla, E.; Kellner, U.; Eckstein, A.; Zrenner, E.; Wissinger, B. RDS/peripherin gene mutations are frequent causes of central retinal dystrophies. Journal of Medical Genetics **1997**, 34, 620–626. §: Meins, M.; Grüning, G.; Blankenagel, A.; Krastel, H.; Reck, B.; Fuchs, S.; Schwinger, E.; Gal, A. Heterozygous ‘null allele’ mutation in the human peripherin/RDS gene, Human Molecular Genetics, Issue, **1993**, 2, 2181–2182. ^: National Center for Biotechnology Information. ClinVar; [VCV000861236.3], https://www.ncbi.nlm.nih.gov/clinvar/variation/VCV000861236.3 (accessed on 28 July 2022).

## Data Availability

Data available from authors.

## Supplementary Materials

The following supporting information can be downloaded at: https://www.mdpi.com/article/10.3390/diagnostics12081851/s1, Figure S1: Multipanel family’s pedigree.

## References

[1] Pontikos N., Arno G., Jurkute N., Schiff E., Ba-Abbad R., Malka S., Gimenez A., Georgiou M., Wright G., Armengol M. (2020). Genetic basis of inherited retinal disease in a molecularly characterized cohort of over 3000 families from the United Kingdom. Ophthalmology.

[2] Manes G., Guillaumie T., Vos W.L., Devos A., Audo I., Zeitz C., Marquette V., Zanlonghi X., Defoort-Dhellemmes S., Puech B. (2015). High prevalence of PRPH2 in autosomal dominant retinitis pigmentosa in france and characterization of biochemical and clinical features. Am. J. Ophthalmol..

[3] Arikawa K., Molday L.L., Molday R.S., Williams D.S. (1992). Localization of peripherin/rds in the disk membranes of cone and rod photoreceptors: Relationship to disk membrane morphogenesis and retinal degeneration. J. Cell Biol..

[4] Molday R.S., Hicks D., Molday L. (1987). Peripherin. A rim-specific membrane protein of rod outer segment discs. Invest. Ophthalmol. Vis. Sci..

[5] Charrin S., Jouannet S., Boucheix C., Rubinstein E. (2014). Tetraspanins at a glance. J. Cell Sci..

[6] Zimmerman B., Kelly B., McMillan B.J., Seegar T., Dror R.O., Kruse A.C., Blacklow S.C. (2016). Crystal Structure of a Full-Length Human Tetraspanin Reveals a Cholesterol-Binding Pocket. Cell.

[7] Boon C.J., den Hollander A.I., Hoyng C.B., Cremers F.P., Klevering B.J., Keunen J.E. (2008). The spectrum of retinal dystrophies caused by mutations in the peripherin/RDS gene. Prog. Retin. Eye Res..

[8] Poloschek C.M., Bach M., Lagrèze W.A., Glaus E., Lemke J.R., Berger W., Neidhardt J. (2010). ABCA4 and ROM1: Implications for Modification of the PRPH2-Associated Macular Dystrophy Phenotype. Investig. Ophthalmol. Vis. Sci..

[9] Reeves M.J., Goetz K.E., Guan B., Ullah E., Blain D., Zein W.M., Tumminia S.J., Hufnagel R.B. (2020). Genotype-phenotype associations in a large PRPH2-related retinopathy cohort. Hum. Mutat..

[10] Zulliger R., Conley S.M., Mwoyosvi M.L., Al-Ubaidi M.R., Naash M.I. (2018). Oligomerization of Prph2 and Rom1 is essential for photoreceptor outer segment formation. Hum. Mol. Genet..

[11] Coco-Martin R.M., Sanchez-Tocino H.T., Desco C., Usategui-Martín R., Tellería J.J. (2020). PRPH2-Related Retinal Diseases: Broadening the Clinical Spectrum and Describing a New Mutation. Genes.

[12] Albertos-Arranz H., Sánchez-Sáez X., Martínez-Gil N., Pinilla I., Coco-Martin R.M., Delgado J., Cuenca N. (2021). Phenotypic Differences in a PRPH2 Mutation in Members of the Same Family Assessed with OCT and OCTA. Diagnostics.

[13] Michaelides M., Holder G.E., Bradshaw K., Hunt D.M., Moore A.T. (2005). Rod dystrophy, intrafamilial variability, and incomplete penetrance associated with the R172W mutation in the peripherin/RDS gene. Ophthalmology.

[14] Vaclavik V., Tran H.V., Gaillard M.C., Schorderet D.F., Munier F.L. (2012). Pattern dystrophy with high intrafamilial variability associated with Y141C mutation in the peripherin/RDS gene and successful treatment of subfoveal CNV related to multifocal pattern type with anti-VEGF (ranibizumab) intravitreal injections. Retina.

[15] Leroy B.P., Kailasanathan A., De Laey J.J., Black G.C., Manson F.D. (2006). Intrafamilial phenotypic variability in families with RDS mutations: Exclusion of ROM1 as a genetic modifier for those with retinitis pigmentosa. Br. J. Ophthalmol..

[16] Palma M., Martin D., Salles M.V., Motta F., Abujamra S., Sallum J. (2019). Retinal dystrophies and variants in PRPH2. Arq. Bras. Oftalmol..

[17] Conley S.M., Naash M.I. (2014). Gene therapy for PRPH2-associated ocular disease: Challenges and prospects. Cold Spring Harb. Perspect. Med..

[18] Sullivan L.S., Bowne S.J., Birch D.G., Hughbanks-Wheaton D., Heckenlively J.R., Lewis R.A., Garcia C.A., Ruiz R.S., Blanton S.H., Northrup H. (2006). Prevalence of disease-causing mutations in families with autosomal dominant retinitis pigmentosa: A screen of known genes in 200 families. Invest. Ophthalmol. Vis. Sci..

[19] Falsini B., Placidi G., De Siena E., Chiurazzi P., Minnella A.M., Savastano M.C., Ziccardi L., Parisi V., Iarossi G., Percio M. (2022). Genetic characteristics of 234 Italian patients with macular and cone/cone-rod dystrophy. Sci. Rep..

[20] Oishi A., Fujinami K., Mawatari G., Naoi N., Ikeda Y., Ueno S., Kuniyoshi K., Hayashi T., Kondo H., Mizota A. (2021). Genetic and Phenotypic Landscape of PRPH2-Associated Retinal Dystrophy in Japan. Genes.

[21] Coussa R.G., Chakarova C., Ajlan R., Taha M., Kavalec C., Gomolin J., Khan A., Lopez I., Ren H., Waseem N. (2015). Genotype and Phenotype Studies in Autosomal Dominant Retinitis Pigmentosa (adRP) of the French-Canadian Founder Population. Invest. Ophthalmol. Vis. Sci..

[22] Marmor M.F., Fulton A.B., Holder G.E., Miyake Y., Brigell M., Bach M., International Society for Clinical Electrophysiology of Vision (2009). ISCEV Standard for full-field clinical electroretinography (2008 update). Doc. Ophthalmol..

[23] McCulloch D.L., Marmor M.F., Brigell M.G., Hamilton R., Holder G.E., Tzekov R., Bach M. (2015). ISCEV Standard for full-field clinical electroretinography (2015 update). Doc. Ophthalmol..

[24] Hood D.C., Bach M., Brigell M., Keating D., Kondo M., Lyons J.S., Marmor M.F., McCulloch D.L., Palmowski-Wolfe A.M., International Society for Clinical Electrophysiology of Vision (2012). ISCEV standard for clinical multifocal electroretinography (mfERG) (2011 edition). Documenta ophthalmologica. Adv. Ophthalmol..

[25] Ziccardi L., Cioffi E., Barbano L., Gioiosa V., Falsini B., Casali C., Parisi V. (2021). Macular Morpho-Functional and Visual Pathways Functional Assessment in Patients with Spinocerebellar Type 1 Ataxia with or without Neurological Signs. J. Clin. Med..

[26] Ziccardi L., Parisi V., Picconi F., Di Renzo A., Lombardo M., Frontoni S., Parravano M. (2018). Early and localized retinal dysfunction in patients with type 1 diabetes mellitus studied by multifocal electroretinogram. Acta Diabetol..

[27] Parisi V., Ziccardi L., Costanzo E., Tedeschi M., Barbano L., Manca D., Di Renzo A., Giorno P., Varano M., Parravano M. (2020). Macular Functional and Morphological Changes in Intermediate Age-Related Maculopathy. Invest. Ophthalmol. Vis. Sci..

[28] Richards S., Aziz N., Bale S., Bick D., Das S., Gastier-Foster J., Grody W.W., Hegde M., Lyon E., Spector E. (2015). Laboratory Quality Assurance Committee. Standards and guidelines for the interpretation of sequence variants: A joint consensus recommendation of the American College of Medical Genetics and Genomics and the Association for Molecular Pathology. Genet. Med. Off. J. Am. Coll. Med. Genet..

[29] Fakin A., Zupan A., Glavač D., Hawlina M. (2012). Combination of retinitis pigmentosa and hearing loss caused by a novel mutation in PRPH2 and a known mutation in GJB2: Importance for differential diagnosis of Usher syndrome. Vis. Res..

[30] Xu J., Li K., Zheng B., Dai H. (2021). Treatment and longitudinal follow-up of CNV associated with pattern dystrophy with novel PRPH2 variant. Ophthalmic Genet..

[31] Lee C.S., Leys M. (2020). A Family Affected by Novel C213W Mutation in PRPH2: Long-Term Follow-Up of CNV Secondary to Pattern Dystrophy. Ophthalmic Surg. Lasers Imaging Retin..

[32] Buccitelli C., Selbach M. (2020). mRNAs, proteins and the emerging principles of gene expression control. Nat. Rev. Genet..

[33] Campello L., Singh N., Advani J., Mondal A.K., Corso-Díaz X., Swaroop A. (2021). Aging of the Retina: Molecular and Metabolic Turbulences and Potential Interventions. Annu. Rev. Vis. Sci..

[34] Arnold J.J., Sarks J.P., Killingsworth M.C., Kettle E.K., Sarks S.H. (2003). Adult vitelliform macular degeneration: A clinicopathological study. Eye.

[35] Campos S.H., Forjaz V., Kozak L.R., Silva E., Castelo-Branco M. (2005). Quantitative phenotyping of chromatic dysfunction in best macular dystrophy. Arch. Ophthalmol..

[36] Ba-Abbad R., Robson A.G., Yap Y.C., Moore A.T., Webster A.R., Holder G.E. (2014). Prph2 mutations as a cause of electronegative ERG. Retina.

[37] Downes S.M., Fitzke F.W., Holder G.E., Payne A.M., Bessant D.A., Bhattacharya S.S., Bird A.C. (1999). Clinical features of codon 172 RDS macular dystrophy: Similar phenotype in 12 families. Arch Ophthalmol..

[38] Duncan J.L., Talcott K.E., Ratnam K., Sundquist S.M., Lucero A.S., Day S., Zhang Y., Roorda A. (2011). Cone structure in retinal degeneration associated with mutations in the peripherin/RDS gene. Investig. Ophthalmol. Vis. Sci..

[39] Schatz P., Abrahamson M., Eksandh L., Ponjavic V., Andréasson S. (2003). Macular appearance by means of OCT and electrophysiology in members of two families with different mutations in RDS (the peripherin/RDS gene). Acta Ophthalmol. Scand..

[40] Falfoul Y., Matri K.E., Habibi I., Halouani S., Chebil A., Schorderet D., El Matri L. (2021). OCT-angiography assessing quiescent and active choroidal neovascularization in retinitis pigmentosa associated with PRPH2 pathogenic variant. Eur. J. Ophthalmol..

[41] Duncker T., Tsang S.H., Woods R.L., Lee W., Zernant J., Allikmets R., Delori F.C., Sparrow J.R. (2015). Quantitative Fundus Autofluorescence and Optical Coherence Tomography in PRPH2/RDS- and ABCA4-Associated Disease Exhibiting Phenotypic Overlap. Investig. Ophthalmol. Vis. Sci..

