# Supplementary figures and images for: Multimodal Study of PRPH2 Gene-Related Retinal Phenotypes
Source: Diagnostics (Basel). 2022 Jul 31;12(8):1851. doi: 10.3390/diagnostics12081851 (PMC9406607; doi:10.3390/diagnostics12081851)

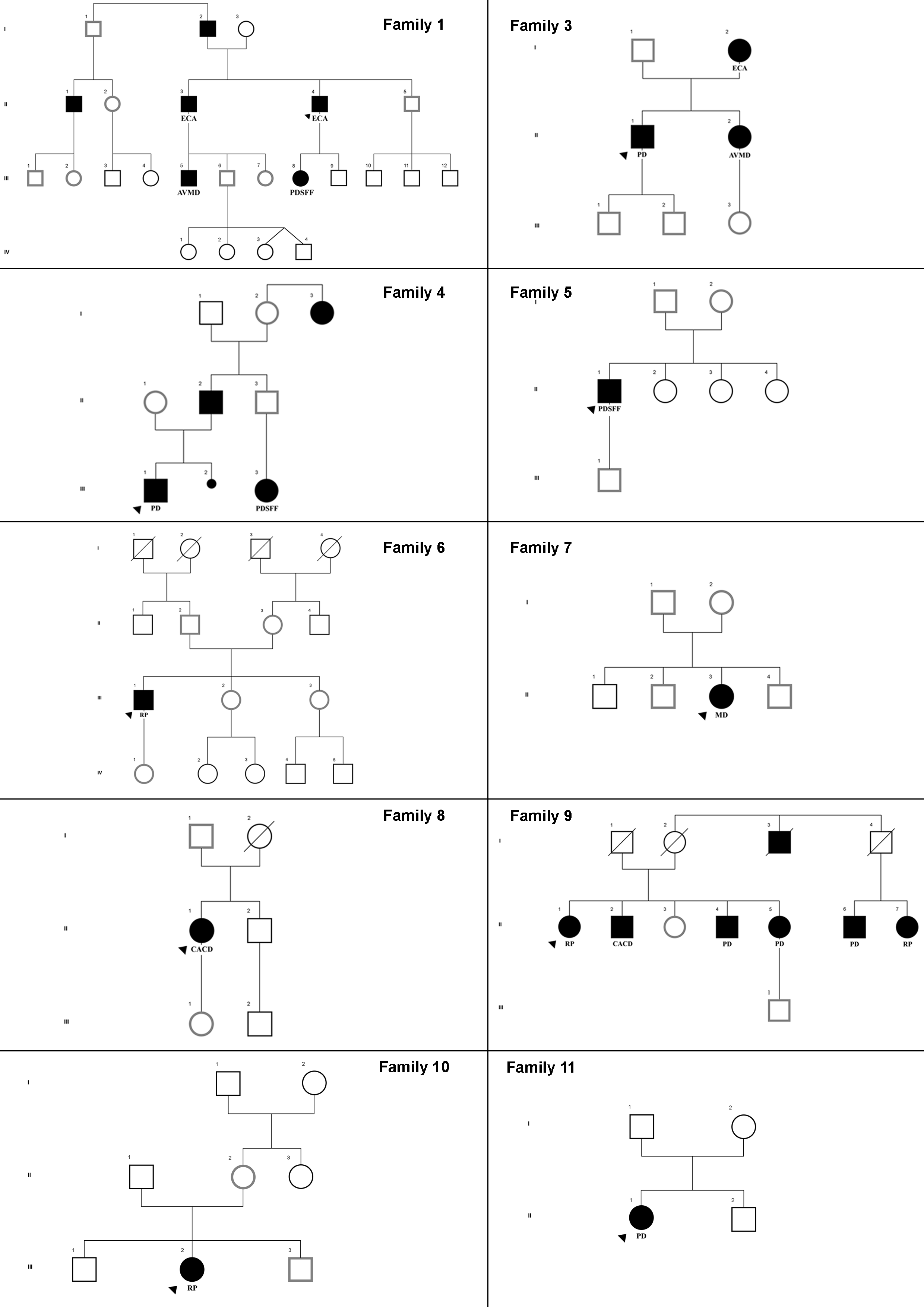

Supplement: Supplementary file 1 [file diagnostics-12-01851-s001.zip › diagnostics-1828913-supplementary.tif]
